# Endometrial stromal cell miR-19b-3p release is reduced during decidualization implying a role in decidual-trophoblast cross-talk

**DOI:** 10.3389/fendo.2023.1149786

**Published:** 2023-03-16

**Authors:** Ellen Menkhorst, Teresa So, Kate Rainczuk, Siena Barton, Wei Zhou, Tracey Edgell, Evdokia Dimitriadis

**Affiliations:** ^1^ Embryo Implantation Laboratory, Department of Obstetrics and Gynecology, The University of Melbourne, Parkville, VIC, Australia; ^2^ Gynecology Research Centre, The Royal Women’s Hospital, Parkville, VIC, Australia; ^3^ Centre for Reproductive Health, Hudson Institute of Medical Research, Clayton, VIC, Australia

**Keywords:** decidua, decidualization, microRNA release, miR-19b-3p, trophoblast, early pregnancy loss

## Abstract

**Introduction:**

A healthy pregnancy requires successful blastocyst implantation into an adequately prepared or ‘receptive’ endometrium. Decidualization of uterine endometrial stromal fibroblast cells (hESF) is critical for the establishment of a healthy pregnancy. microRNAs (miRs) are critical regulators of cellular function that can be released by a donor cell to influence the physiological state of recipient cells. We aimed to determine how decidualization affects hESF miR release and investigated the function of one decidualization regulated miR, miR-19b-3p, previously shown to be associated with recurrent pregnancy loss.

**Method:**

miR release by hESF was determined by miR microarray on culture media from hESF decidualized *in vitro* for 3 and 14 days by treatment with oestradiol and medroxyprogesterone acetate. Cellular and whole endometrial/decidual tissue miR expression was quantified by qPCR and localized by in situ hybridization. The function of miR-19b-3p in HTR8/Svneo trophoblast cells was investigated using real time cell analysis (xCELLigence) and gene expression qPCR.

**Results:**

From our miR screen we found that essentially all hESF miR release was reduced following in vitro decidualization, significantly so for miR-17-5p, miR-21-3p, miR-34c-3p, miR-106b-5p, miR-138-5p, miR-296-5p, miR-323a-3p, miR-342-3p, miR-491-5p, miR-503-5p and miR-542-5p. qPCR demonstrated that miR-19b-3p, 181a-2-3p and miR-409-5p likewise showed a significant reduction in culture media following decidualization but no change was found in cellular miR expression following decidualization. *In situ* hybridization localized miR-19b-3p to epithelial and stromal cells in the endometrium and qPCR identified that miR-19b-3p was significantly elevated in the cycling endometrium of patients with a history of early pregnancy loss compared to normally fertile controls. Functionally, overexpression of miR-19b-3p significantly reduced HTR8/Svneo trophoblast proliferation and increased HOXA9 expression.

**Discussion:**

Our data demonstrates that decidualization represses miR release by hESFs and overexpression of miR-19b-3p was found in endometrial tissue from patients with a history of early pregnancy loss. miR-19b-3p impaired HTR8/Svneo proliferation implying a role in trophoblast function. Overall we speculate that miR release by hESF may regulate other cell types within the decidua and that appropriate release of miRs by decidualized hESF is essential for healthy implantation and placentation.

## 1 Introduction

A healthy pregnancy requires successful blastocyst implantation into an adequately prepared or ‘receptive’ endometrium. Decidualization of human uterine human uterine endometrial stromal fibroblast (hESF) is critical for the establishment of a healthy pregnancy (1, 2); impaired decidualization is associated with poor pregnancy outcomes including recurrent early pregnancy loss and preeclampsia (3–5). Decidualization is initiated post-ovulation by corpus luteum-secreted progesterone and involves the reprogramming of hESF, including significant phenotypic and functional changes: hESF become rounded, highly secretory and with altered extellular matrix expression (1). In women, decidualization begins each menstrual cycle regardless of the presence of a functional blastocyst (1). Decidual cells interact with the implanting blastocyst to facilitate implantation and placentation: they regulate extravillous trophoblast (EVT) proliferation, migration and invasion (6–8), shield the conceptus from environmental stress signals (1), regulate the recruitment and differentiation of the uterine-resident immune cell population (9–11) and are thought to ‘sense’ the quality of the conceptus, facilitating rejection of incompetent embryos (12, 13).

microRNAs (miRs) are critical regulators of cellular function and have been most intensively investigated in cancer, where they regulate metastasis, angiogenesis and inflammation (14, 15). miRs can also act as ‘hormones’ – donor cells (which release the miR) can influence the physiological state of recipient cells (cells which take up the miR) over short (cell to neighboring cell) and long (effects on a different organ) distances (15, 16). In pregnancy, miRs are produced by cells within the decidua (decidual cells, leucocytes and endothelial cells) (17) and placental villous trophoblast (18). miR expression is altered in the decidua of early pregnancy loss compared to healthy pregnancies (18).

Less is known about miRs during endometrial remodelling. *In vitro*, decidual cellular miRs regulate decidualization (19), however little known about how secreted endometrial miRs may regulate other cells within the decidua including trophoblast. We aimed to determine how decidualization affected hESF miR release and determine the expression and function of one hESF released miR, miR-19b-3p, previously associated with recurrent early pregnancy loss (20).

## 2 Methods

### 2.1 Primary tissue collection

This study followed the NHMRC guidelines for ethical conduct in human research. Ethics approvals for this study were provided by The Royal Women’s Hospital and Monash Health Human Research and Ethics Committees (#90317B, #06014C and #03066B). Written and informed consent was obtained from each participant.

Endometrial biopsies were collected by dilatation and curettage (n=26 women; **Table 1**). Five biopsies were used for decidualization experiments (1 with history of early pregnancy loss), 3 for *in situ* (2 with history of early pregnancy loss) and 18 for RNA extraction (12 fertile, 6 with a history of early pregnancy loss). The women had no hormonal treatment for ≥ 3 months before tissue collection.

**Table 1:** Characteristics of non-pregnant participants.

	Normally fertile (n=17)	Pregnancy Loss (n=9)
Maternal age (years)^a^	35.5+/-1.5 (26-46)	37.3+/-1.3 (33-41)
Gravidity^b^	2.3+/-0.2 (1-3)	3.7+/-1.2 (2-6)
Parity median^c^	2.0 (0-3)	0.0 (0-1)
Number of previous losses^d^	0	3.3+/-0.9 (2-5)

Data provided as the mean ± SEM; range given in brackets.

a data unavailable for 3 fertile patients; 2 pregnancy loss patients.

b data unavailable for 6 fertile patients; 6 pregnancy loss patients.

c data unavailable for 5 fertile patients; 4 pregnancy loss patients.

d data unavailable for 6 fertile patients; 6 pregnancy loss patients – defined as ‘multiple’ only.

First trimester products of conception were collected following elective termination of pregnancy by evacuation for psychosocial reasons (n=4; amenorrhea 6-11 weeks). Term placental villous and decidual tissue was donated by healthy women following spontaneous labor at term (>37 weeks; n=4).

Serum was collected from women aged >18 years (n=5/group) attending an IVF clinic, who had successful pregnancies following IVF and those who had repeated pregnancy loss following IVF. Serum was collected from women undergoing oocyte collection, two days after induction of ovulation by human chorionic gonadotrophin. Subsequent details of outcomes of embryo transfer in the same cycle were recorded.

### 2.2 Cell culture

All cells were cultured at 37°C in a 5% CO_2_ humidified culture incubator. hESF were maintained in DMEM/F12 (Gibco, Thermo Fisher Scientific, Inc.) plus 10% charcoal stripped Fetal Bovine serum (FBS; Gibco, Thermo Fisher Scientific, Inc.) and 1% antibiotics (penicillin, streptomycin, amphoceterin B; Gibco, Thermo Fisher Scientific, Inc.). HTR8/SVneo cells (CRL-3271) were from the ATCC and cultured with RPMI (Gibco, Thermo Fisher Scientific, Inc.) plus 10% heat inactivated FBS (Gibco, Thermo Fisher Scientific, Inc.).

### 2.3 Decidualization

hESF were isolated using collagenase digestion and filtration as previously described (21), resulting in a 97% stromal fibroblast population (22). hESF were decidualized as previously described (21) by treatment for 14 days with oestradiol (E, 10^-8^M; Sigma) and medroxyprogesterone acetate (MPA, 10^-7^M; Sigma) in DMEM/F12 containing 2% charcoal stripped FBS and 1% antibiotics. The media was refreshed every 2-3 days, on a Monday, Wednesday and Friday. Cells and culture media were collected on Day 3 and Day 14, both after 72h of culture. Cells were pelleted by centrifugation at 500x*g* then snap-frozen. Culture media was centrifuged at 500x*g* for 5 minutes to pellet cell debris then the supernatant snap-frozen.

### 2.4 Prolactin ELISA

PRL secretion by decidualized hESF (culture media collected on days 3 and 14) was quantified by ELISA as per the manufacturer’s instructions (DuoSet kit #DY682, R&D systems) (23).

### 2.5 RNA isolation

*Decidual culture media:* RNA was isolated from 200uL culture media collected on Day 3 and Day 14 of culture and media only control using the RNeasy Micro Kit (Qiagen) according to the manufacturer’s instructions.

*hESF & HTR8/Svneo cells, endometrial and decidual tissue:* RNA extraction was performed as previously described using Tri Reagent according to the manufacturer's instructions (Sigma-Aldrich, Merck).

*Serum:* RNA extraction (from 250uL serum) was performed using the TRIzol LS reagent (Ambion, Life Technologies) as per the manufacturer’s instructions.

Genomic DNA was removed from isolated RNA using the DNAfree kit (Ambion; Thermo Fisher Scientific, Inc.) according to the manufacturer’s protocol. A spectrophotometer (Nanodrop Technologies; Thermo Fisher Scientific, Inc.), was used at an absorbance ratio of 260/280 nm to analyze RNA sample concentration, yield and purity.

### 2.6 microRNA array

cDNA synthesis was performed using the miRCURY LNA™ Universal RT microRNA PCR system (Qiagen) and microRNA PCR Human Panel (I) as previously described (24). cDNA products diluted 60-fold were plated on the microRNA PCR Human Panel (I) plate and qPCR was performed using a 7900HT thermocycler (Applied Biosystems) using the recommended parameters (Qiagen). Raw CT values were normalized (ΔCT) to the average of the control wells (UniSP3) on the plate, then ΔΔCT calculated by normalizing the ΔCT to the average of the day 3 samples for each gene (**Supplementary Table 1**). A media only control was run to enable exclusion of miRs present in the treatment media.

### 2.7 miR RT-qPCR

cDNA was synthesized from 10ng total RNA using the TaqMan reverse transcription kit (Applied Biosystems; Thermo Fisher Scientific, Inc), and specific TaqMan miR primer sets (cat no. #4427975; miR-19b-3p #000396; miR-181a-2-3p #002317; miR-409-5p #002331; rnU6, #001973; Applied Biosystems; Thermo Fisher Scientific, Inc.) on the Veriti 7 fast block real-time qPCR system (Applied Biosystems). miR qPCR was performed in triplicate (final reaction volume, 10 μl) in 384-well micro- optical plates (Applied Biosystems; Thermo Fisher Scientific, Inc.) on the ABI 7900HT fast block or Viia 7 qPCR systems (Applied Biosystems; Thermo Fisher Scientific, Inc.). A template-free negative control and RNase-free water only was added for each run. The qPCR conditions were: 95°C for 10 min and 40 cycles of 95°C for 15s followed by 60°C for 1 min. Relative expression levels were calculated as per the manufacturer’s instructions using the comparative cycle threshold method (ΔΔCT).

### 2.8 mRNA RT-qPCR

Total RNA (250ng) was reverse transcribed using Superscript III (Invitrogen) (0.5 µL per reaction) as previously described (25). qPCR was performed as previously described (25) using Power SYBR Green master mix (Applied Biosystems) on the Veriti 7 fast block real-time qPCR system (Applied Biosystems). Primer sequences are as follows: *18s* Fwd: 5`GATCCATTGGAGGGCAAGTCT3`, Rev: 5`CCAAGATCCACCTACGAGCTT3`; Fwd: *HOXA9* 5`TACGTGGACTCGTTCCTGCT3`, Rev: 5`CGTCGCCTTGGACTGGAAG3`; *PTEN* Fwd: 5`TCCATCCTGCAGAAGAAGCC3`, Rev: 5`AGGATATTGTGCAACTCTGCAA3`; (Sigma-Aldrich). A template-free negative control in the presence of primers and RNase-free water only negative controls were added for each run. The qPCR conditions were: 95°C for 10 min and 40 cycles of 95°C for 15s followed by 60°C for 1 min. Relative expression levels (normalized to *18s* ribosomal RNA) were calculated as per the manufacturer’s instructions using the comparative cycle threshold method (ΔΔCT).

### 2.9 *In situ* hybridization

*In situ* hybridization was performed as previously described (26). Briefly, 4 μm thickness endometrial sections were deparaffinized and rehydrated in xylene, neat ethanol, 96% ethanol, and 70% ethanol and then placed in PBS Proteinase K (15 μg/mL) digestion was performed at 37°C for 15 min. Following PBS wash, 100 nM miR-19b-3p detection probe (#339111, YD00619863-BCG; Qiagen) or scramble control probe (cat no. #339111 YD00699004-BCG) was applied to sections and placed in a 60°C incubator for 1 h. Slides were then washed in 5x sodium-saline citrate (SSC), 1x SSC and 0.2x SSC buffers at 60°C for 5 min, and 0.2x SSC at room temperature (RT) for 5 min, then placed in PBS. Blocking solution of 10% CAS block (008120, Thermo), 2% sheep serum, 1% bovine serum albumin (BSA) in PBS-Tween (T) was applied to sections and incubated at RT for 15 min. After incubation, sections were treated with anti-DIG-fluorescein 1:50 in 0.5% BSA/PBS at RT for 1 h. Following additional washes in PBS-T, sections were counterstained with DAPI to indicate the cell nuclei (blue). Sections were visualized using Olympus BX63 fluorescence microscope and cellSense software. All images were taken under the same exposure and settings.

### 2.10 Real time cell analysis

The real-time cell analyser (RTCA) MP xCELLigence instrument (ACEA Biosciences; Agilent Technologies GmbH) was used to interrogate the effect of miR-19b-3p on HTR8/Svneo adhesion and proliferation. HTR8/Svneo were transfected with 100nM miR-19b-3p mimic (cat no. 339173 YM00470545-ADB) or negative control (cat no. 339173 YM00479902-ADB) using Lipofectamine RNAiMAX (13778100, Thermo Fisher) and Opti-MEM medium (11524456, Fisher) following manufacturer’s instructions for 72 h. After transfection cells were seeded into E-plate 96 (ACEA Biosciences; Agilent Technologies GmbH) at ~10,000 cells/well in RPMI supplemented with 5% FCS. Data was collected ever 15 minutes for a total of 96h.

### 2.11 Statistical analysis

Statistical analyses were performed using GraphPad Prism 9.5.0. Paired t-tests, one-way ANOVA and repeated measures ANOVA were performed. All data is presented as mean ± SEM. P<0.05 was considered statistically significant.

## 3 Results

### 3.1 hESF miR release is repressed by decidualization

**Figure 1:**
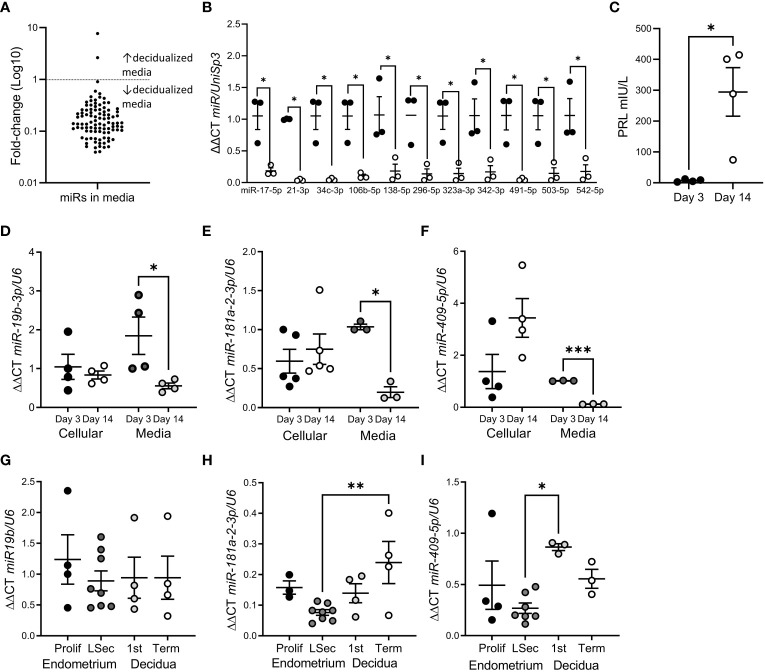
miR release was reduced following hESF decidualization. **(A)** Fold-change of all miRs identified in hESF culture media by microarray from Day 3 to Day 14. **(B)** miRs with significantly reduced levels in hESF culture media between Day 3 and Day 14. **(C)**. Prolactin (PRL) secretion by hESF on Day 3 and Day 14 of decidualization. **(D–F)**. qPCR of miR-19b-3p **(D)**, miR-181a-2-3p **(E)** and miR-409-5p **(F)** in hESF cells and culture media on Day 3 and Day 14 of decidualization. **(G–I)**. qPCR of miR-19b-3p **(G)**, miR-181a-2-3p **(H)** and miR-409-5p **(I)** in whole tissue biopsies collected during the proliferative (prolif) and late secretory (LSec) stages of the menstrual cycle and 1^st^ trimester and term decidua. Data shows mean ± SEM; *P<0.05; **P<0.01; ***P<0.001; **(C–F)**, paired t-test; **(G–I)**, one-way ANOVA.

We identified 98 miRs released by hESF into the culture media (**Figure 1A**; **Supplementary Table 1**). The most highly expressed miRs were miR-125b-5p, -23a-3p and let-7b-5p. miR release into the culture media was highly repressed following *in vitro* hESF decidualization (**Figure 1A**): 11 miRs showed a significant reduction at Day 14 of *in vitro* decidualization (**Figure 1B**). Decidualization was confirmed by PRL secretion (**Figure 1C**).

To confirm the array data, we investigated the expression of 3 different miRs in hESF matched cellular and culture media RNA (**Figures 1D–F**). Interestingly, although the cellular levels of miRs-19b-3p, -181a-2-3p and -409-5p were not altered by decidualization, miR concentration in culture media was significantly reduced (**Figures 1D–F**). In contrast when we investigated whether decidualization altered miR-19b-3p, -181a-2-3p or -409-5p expression in whole endometrial tissue biopsies (non-decidualized:proliferative endometrium; decidualized: late secretory endometrium, 1^st^ trimester or term decidua) (**Figures 1G–I**) we found no difference in expression between non-decidualized and decidualized tissue, although miR-181a-2-3p was significantly elevated in term decidua compared to late secretory endometrium (**Figure 1H**) and miR-409-5p was significantly elevated in 1^st^ trimester decidua compared to late secretory endometrium (**Figure 1I**).

### 3.2 Endometrial miR-19b-3p is increased in women with a history of early pregnancy loss

**Figure 2:**
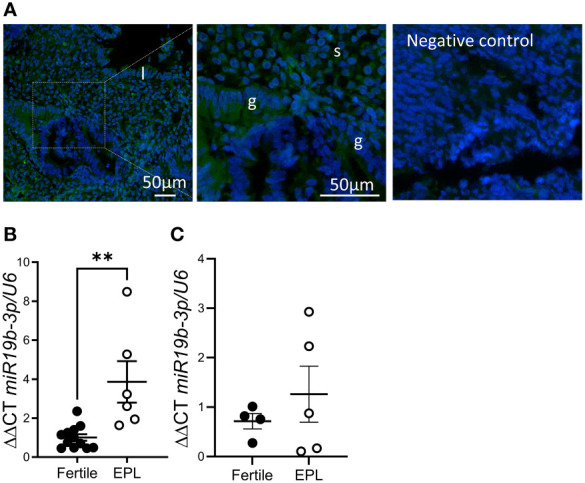
miR-19b-3p expression is elevated in endometrium from women with a history of early pregnancy loss. **(A)** *In situ* hybridization of miR-19b-3p in endometrium. Localization of miR-19b-3p indicated by green fluorescent staining. DAPI (blue) counterstaining identifies nuclei. **(B)** qPCR of miR-19b-3p in endometrium from fertile patients and patients with a history of early pregnancy loss (EPL). **(C)** qPCR of miR-19b-3p in serum from fertile patients and patients with a history of early pregnancy loss. g, glandular epithelium; l, luminal epithelium; s, stroma; Data shows mean ± SEM; **P<0.01; **(B, C)**, paired t-test.

*In situ* hybridization of cycling endometrial tissue biopsies localized miR-19b-3p to most cell types in the endometrium, although endometrial glandular epithelial cell expression was variable even within adjacent glands (**Figure 2A**). Using qPCR, we found that expression miR-19b-3p in endometrial tissue biopsies was significantly increased in patients with a history of early pregnancy loss compared to fertile controls (**Figure 2B**). This increase was not found in serum from women undergoing IVF with a history of repeated early pregnancy loss (**Figure 2C**).

### 3.3 miR-19b-3p reduces HTR8/Svneo trophoblast proliferation

**Figure 3:**
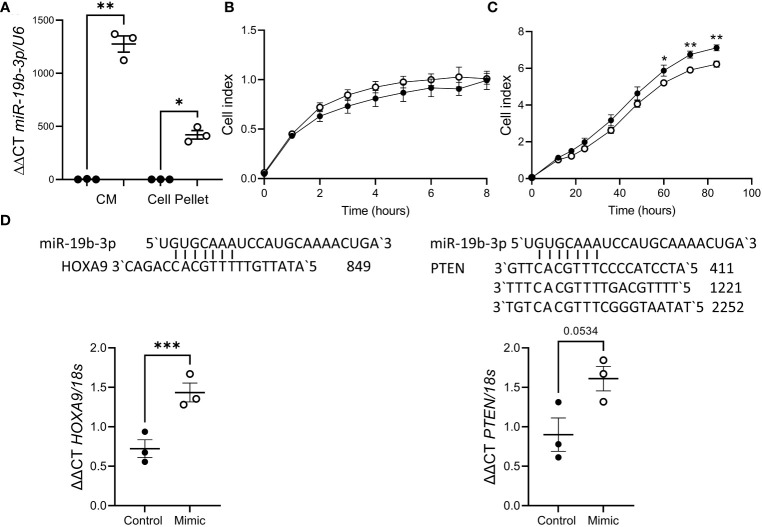
miR-19b-3p overexpression in HTR8/Svneo cells impaired proliferation. **(A)** Treatment with miR-19b-3p mimic (○) significantly increased miR-19b-3p levels in HTR8/Svneo culture media (CM) and cell pellet compared to scramble control (●). **(B)** miR-19b-3p mimic had no effect on HTR8/Svneo adhesion (n=3/group). **(C)** miR-19b-3p mimic significantly reduced HTR8/Svneo proliferation after 60h (n=3/group). **(D)** miR-19b-3p mimic significantly increased *HOXA9* expression but had no effect on *PTEN*. Alignment of miR-19b-3p and the 3`UTR of HOXA9 and PTEN is also shown. Data shows mean ± SEM; *P<0.05; **P<0.01; ***P<0.001; **(A–C)**, repeated measures ANOVA; **(D)**, paired t-test.

As impaired decidualization is associated with recurrent pregnancy loss and miR-19b-3p release was suppressed by decidualization, we investigated the effect of miR-19b-3p on trophoblast function using the HTR8/Svneo cell line. HTR8/Svneo transfected with miR-19b-3p mimic showed elevated miR-19b-3p expression in the cell pellet (**Figure 3A**), suggesting that miR-19b-3p is taken up from the media. Using a Real-Time Cell Analysis system (xCELLigence) we found there was no effect of miR-19b-3p on HTR8/Svneo adhesion (**Figure 3B**) but after 60h miR-19b-3p significantly inhibited HTR8/Svneo proliferation compared to control (**Figure 3C**). We investigated whether transfection with the miR-19b-3p affected HTR8/Svneo production of predicted miR-19b-3p targets (27, 28): miR-19b-3p increased *HOXA9* mRNA but had no effect on *PTEN* mRNA (**Figure 3D**).

## 4 Discussion

Here we showed for the first time that decidualization was associated with a global repression of miR release by hESFs. We found that endometrial tissue collected from women with a history of early pregnancy loss had significantly higher mir-19b-3p production conmpared to fertile controls and transfection of miR-19b-3p mimic to HTR8/Svneo trophoblast cells significantly impaired cell proliferation and increased *HOXA9* mRNA production.

Our observation that global miR release was reduced in decidualized hESF is striking. Released miRs can be transferred to another cell, triggering actions in target cells (15). Certainly, decidualized cell secretions promote decidualization of surrounding stromal cells (1), regulate uterine-resident lympocyte recruitment and differentiation (29) and promote trophoblast invasion (8, 30, 31). We hypothesize hESF-released miRs would be taken up by surrounding cells (eg. other decidual cells, trophoblast, immune and endothelial cells) and our data suggests that decidualization may release these other cells from hESF mediated control. We did not investigate the mechanism by which this repression in miR release occurs, however extracellular vesicle production is increased following decidualization with cAMP (32), suggesting that there may be a change in other methods of release (eg argonaute proteins). Whether argonaute proteins in hESF are regulated by decidualization has not been investigated.

It was somewhat surprising that we didn’t see a change in cellular miRs using this *in vitro* model as have been seen in other models that investigated only cellular miRs following *in vitro* decidualization, including miR-181a (downregulated 3-fold) and miR-409-5p (upregulated 2.3-fold) (17). We saw a non-significant trend to increased miR-409-5p cellular expression and a significant increase in production in the 1^st^ trimester decidua compared to late secretory phase endometrium. To exclude the direct effect of oestradiol or MPA on miR release in this study we collected cells and culture media 3 days after initiating the decidualization treatments. Although there is negligible PRL secretion on day 3, it is possible that alterations to miR production are initiated early in decidualization and we may have seen an effect on miR production if we compared hESF before and after decidualization hormone treatment as is done in other studies.

Collectively, previous studies and our results suggest that dysregulated miR-19b-3p production may be involved in the etiology of recurrent pregnancy loss. We found that miR-19b-3p was significantly elevated in the cycling endometrium of patients with a history of early pregnancy loss and Tian et al. showed that miR-19b-3p is decreased in the placental villous of patients with a history of recurrent early pregnancy loss (20). Furthermore, miR-19b-3p is dysregulated in monocytes from patients with antiphospholipid syndrome (33), an acquired thrombophilia diagnosed in 15-20% of patients with recurrent early pregnancy loss (5).

The function of miR-19b-3p appears highly cell type specific. In trophoblast miR-19b-3p overexpression prevents syncytialization of primary human cytotrophoblasts (34), decreased PTEN production in JEG-3 (20) and here we found miR-19b-3p impaired HTR8/Svneo trophoblast proliferation. In other tissues miR-19b-3p mostly promotes proliferation (35–39). The inhibition of proliferation by miR-19b-3p mimic seen here may be due to the increase in *HOXA9* production also stimulated by the miR-19b-3p mimic: HOXA9 inhibits HTR8/Svneo proliferation (40), migration and invasion (41).

A role for miR-19b-3p in inflammation is also proposed, however again the function of miR-19b-3p in regulating inflammatory responses is not clear. miR-19b-3p increases apoptosis and intracellular reactive oxygen species in endothelial cells (42), enhances Th1/M1 inflammatory responses (43–45) and inhibits Treg differentiation (43), but may also promote M2 polarization (46, 47). That miR-19b-3p may be pro-inflammatory in pregnancy is suggested by elevated levels in maternal plasma of pregnancies with gestational diabetes mellitus (48, 49), and preterm birth (50), both inflammatory conditions. Loss of miR-19b-3p in the endometrium during decidualization therefore may be crucial to promote trophoblast differentiation and maternal tolerance.

In conclusion, we found that *in vitro* decidualization was associated with reduced miR release and that overexpression of miR-19b-3p was found in endometrial tissue from patients with history of early pregnancy loss. Finally, we found that miR-19b-3p impaired HTR8/Svneo proliferation implying a role for this decidual-released miR in trophoblast function. Overall we speculate that decidualization may act to reduce endometrial stromal cell regulation of other cell types within the decidua, particularly trophoblast, enabling healthy implantation and placentation during early pregnancy.

## Data availability statement

The original contributions presented in the study are included in the article/**Supplementary Material**, Further inquiries can be directed to the corresponding author/s.

## Ethics statement

The studies involving human participants were reviewed and approved by Royal Women’s Hospital Research Ethics Committee Monash Health Human Research Ethics Committee. The patients/participants provided their written informed consent to participate in this study.

## Author contributions

EM conception, design, experiments, wrote manuscript. TS experiments. KR experiments. SB experiments. WZ experiments, edited manuscript. TE design, samples, edited manuscript. ED design, edited manuscript. All authors contributed to the article and approved the submitted version.
